# Functional Genomics for the Identification of Modulators of Platelet-Dependent Thrombus Formation

**DOI:** 10.1055/s-0038-1670630

**Published:** 2018-09-10

**Authors:** Elien Vermeersch, Benedicte P. Nuyttens, Claudia Tersteeg, Katleen Broos, Simon F. De Meyer, Karen Vanhoorelbeke, Hans Deckmyn

**Affiliations:** 1 Laboratory for Thrombosis Research, KU Leuven Campus Kulak, Kortrijk, Belgium

**Keywords:** knockout mice, hemostasis, megakaryocytes, thrombosis, transgenic mice

## Abstract

Despite the absence of the genome in platelets, transcription profiling provides important insights into platelet function and can help clarify abnormalities in platelet disorders. The Bloodomics Consortium performed whole-genome expression analysis comparing in vitro–differentiated megakaryocytes (MKs) with in vitro–differentiated erythroblasts and different blood cell types. This allowed the identification of genes with upregulated expression in MKs compared with all other cell lineages, among the receptors BAMBI, LRRC32, ESAM, and DCBLD2. In a later correlative analysis of genome-wide platelet RNA expression with interindividual human platelet reactivity, LLRFIP and COMMD7 were additionally identified. A functional genomics approach using morpholino-based silencing in zebrafish identified various roles for all of these selected genes in thrombus formation. In this review, we summarize the role of the six identified genes in zebrafish and discuss how they correlate with subsequently performed mouse experiments.

## Selection of Novel Players in Thrombus Formation by Transcriptome Analysis

Blood platelets are important mediators of thrombus formation. However, addressing the platelet genome is challenging, as platelets are anucleated cells derived from megakaryocytes (MKs) in the bone marrow. Platelets are not able to perform de novo transcription due to the lack of genomic DNA. However, mRNA from MKs is transferred in an organized way into platelets and platelets are able to exchange mRNA with other cells via mRNA-rich microvesicles. 1 2

Bloodomics Consortium deployed two different strategies to identify novel regulators of platelet function. First, unknown genes with a specific expression on platelets and endothelial cells were revealed to study their associated phenotype. Comparative transcriptome analysis of the major nucleated blood cell elements and in vitro–differentiated MKs and erythroblasts was performed. This allowed identification of 279 genes with upregulated expression exclusively in MKs. 3 The selection was narrowed down to 75 genes by the identification of putative transmembrane domains. As many known MK/platelet-specific membrane proteins are also expressed in endothelial cells, the genes expressed in both MK and resting human umbilical vein endothelial cells (HUVECs) were identified, narrowing down the number genes to 35. Of these 35 genes, 4 genes were selected for further analysis due to their unknown role in hemostasis and thrombosis, interesting protein domains, and human and zebrafish orthology. 3 4 Expression of all four proteins was confirmed in human platelets. 4

First, vessel wall Bone morphogenetic protein and Activin Membrane Bound Inhibitor ( *BAMBI* ) was selected. BAMBI is a known nonsignaling, transmembrane receptor that associates with transforming growth factor (TGF)-β family receptors and functions as a negative modulator of multiple, often opposing, actions of TGF-β. 5 BAMBI is expressed on, among others, human and mouse endothelial cells, where it functions as a regulator of angiogenesis and vascular homeostasis. 6 7 The second selected transcript was leucine-rich repeat containing 32 (LRRC32), also known as glycoprotein A repetitions predominant (GARP). LRRC32 is necessary for the binding of latent TGF-β to the surface of regulatory T cells and its presence has been shown on the surface of platelets in association with latency-associated peptide (LAP). 4 8 9 10 Third, endothelial cell-selective adhesion molecule ( *ESAM* ), a cell adhesion molecule on endothelial cells colocalizing with cell adhesion proteins such as cadherins, catenins, ZO-1, occluding, and claudin-5, and associated with tight junctions in the blood capillaries, was identified for further analysis. 11 12 13 14 ESAM is associated with the platelet α-granule membrane and highly expressed on the platelet surface upon activation. 12 14 15 Lastly, Discoidin, CUB and LCCL domain containing 2 (DCBLD2), also known as endothelial and smooth muscle cell-derived neuropilin-like molecule (ESDN), was selected. The DCBLD2 protein was identified on cultured smooth muscle cells and endothelial cells and its expression was increased on rat carotid arteries after balloon injury 16 and therefore DCBLD2 was suggested to play a role in vascular cell growth. DCBLD2 was shown to associate with VEGFR2 and loss of *Dcbld2* impaired normal vascular development and weakened VEGF-induced VEGFR2 signaling. 17 Next to its presence on the vascular wall, DCBLD2 expression on platelets was confirmed by Western blot. 4 18

The second strategy used by the Bloodomics Consortium to identify novel platelet regulators in hemostasis was to examine a correlation between the expression of genes and platelet function. Goodall et al reported that transcript levels of 63 genes in human platelets correlated with variation in platelet response toward ADP and/or collagen-related peptide. 19 Six out of the 63 genes were selected for an association study, based on the presence of SNPs within the genes that showed a possible association with either myocardial infarction or platelet response. The two genes with SNPs with the lowest *p* -value of association were finally selected. These were LRR-binding FLII interacting protein 1 (LRRFIP1; rs3739038), with SNPs associated with myocardial infarction, and Copper metabolism Murr1 domain containing 7 (COMMD7; rs6141803), with SNPs associated with platelet response. 19 COMMD7 is expressed in a variety of tissues with the highest expression in the lungs and heart, 20 but cell type–specific expression has not been studied. To the best of our knowledge, the expression of COMMD7 in human platelets was reported (without data being shown) only in platelet lysates using Western blot. 21 Within the cell, COMMD7, like other members of the 10-member family of COMMD genes, acts as a repressor of transcription of the NFκB1 gene, a key transcription factor controlling many proinflammatory genes. 20 Also LRRFIP1 plays a role in the regulation of gene transcription where it has been identified as a repressor of transcription of genes such as EGFR, PDGF-α, and TNF-α. 22 23 Suppression of PDGF-α, for example, was observed upon arterial injury in rats, caused by a sustained increase of LRRFIP1 and resulting in decreased smooth muscle cell proliferation. 22 In addition, LRRFIP1 is known as a cytosolic nucleic acid sensor, binding to cytoplasmic dsDNA and dsRNA, resulting in the recruitment and activation of β-catenin, leading to type I interferon production. 24 The expression of LRRFIP1 on human platelets was confirmed via Western blot 19 ; however, for the earlier-mentioned reasons, a role in the anucleate platelet cannot immediately be interfered.

The human platelet transcriptome was later mapped using next-generation RNA sequencing in an elegant study by Rowley et al and the presence of the transcripts of these newly identified genes, postulated to be involved in hemostasis, was demonstrated. 25 To clarify the putative role of all these genes in thrombosis and hemostasis, functional genomic studies were needed, using the zebrafish model as a first rapid screening method.

## The Zebrafish as an Animal Model for Thrombosis

### Hemostatic System of the Zebrafish

An important prerequisite for using the zebrafish model in hemostasis and thrombosis research is that its hemostatic system demonstrates significant similarities to the human one. 26 Zebrafish indeed have plasma coagulation factors, a vascular system, and thrombocytes. 27 Although thrombocytes from zebrafish are highly similar to platelets from mice and humans, the main difference is the presence of nuclei in thrombocytes. Thrombocytes are formed by the differentiation of thrombocyte precursors, whereas platelets are formed by the release of anucleate fragments of MKs. Nevertheless, the main thrombocyte receptors important in adhesion and aggregation are conserved between human and zebrafish. Jagadeeswaran and colleagues could identify the presence of glycoprotein (GP)Ib and integrin αIIβ3 on thrombocytes using immunofluorescent microscopy. 28 Moreover, the thrombin receptors protease-activated receptor (PAR)-1 and PAR-2, the adenosine 5′-diphosphate (ADP) P2Y receptors, and the cyclooxygenase enzymes COX-1 and COX-2 and thromboxane A synthase 1 needed for thromboxane production are also present on the thrombocyte membrane. 26 29 30 On the other hand, the presence of the collagen receptor GPVI has not been demonstrated thus far, but a GPVI-like receptor was identified that signals through its ITAM and knockdown of the gene results in a delay of thrombus formation in the venous and arterial vessels after a laser-induced thrombosis model. 31 Whole blood thrombocyte adhesion and aggregation was performed using the agonists ristocetin, collagen, thrombin, ADP, and arachidonic acid, demonstrating ristocetin-induced thrombocyte adhesion without ATP release and agonist-induced aggregation with ATP release. 28 Taken together, the thrombocyte function in zebrafish is remarkably similar to the platelet function in human. Although the endothelium of the vasculature is not identical to that of humans, several proteins needed for the control of hemostasis are found. The presence of von Willebrand factor (VWF) was confirmed on endothelial cells of zebrafish. 27 32 Full knockdown of the *Vwf* gene created by antisense morpholino oligonucleotides furthermore resulted in a bleeding phenotype in half of the embryos, which confirms that VWF is also important in the hemostatic processes of zebrafish, 32 similar to its role in mice 33 and humans. 34 This, together with the ease of generating knockdown of genes, provides sufficient arguments to use zebrafish as a first functional genomics model.

### Laser-Induced Thrombosis Model in Zebrafish

In the zebrafish laser-induced thrombosis model, thrombus formation in the caudal artery is caused by laser injury of the endothelium. Thrombocytes play a major role in occlusive thrombus formation where both the time to attachment (TTA) and the thrombus surface area (TSA) are the main quantification parameters. 35 Downregulation of the fibrinogen receptor αIIb, as a positive control, resulted in normal thrombocyte adhesion (TTA) but a defective aggregation with a smaller thrombus size (TSA) compared with animals treated with control morpholinos. 4 35 Also, other studies showed that inhibition of the expression of myosin light chain kinase 1a and protein kinase Cα and β resulted in an attenuated thrombus formation compared with controls. 36 37 These results demonstrated that the effects of proteins with a known relevance in human platelet function could be reproduced in experiments using the laser-induced thrombosis model in zebrafish, once more indicating that the model seems well applicable for the investigation of the function of the newly identified genes.

## Functional Genomics in Zebrafish to Reveal the Function of Newly Identified Genes

The transcriptomics study showed lineage-specific expression of *Bambi* , *Lrrc32* , *Esam* , and *Dcbld2* in MK and HUVECs and were the candidate genes encoding receptors with a putative role in hemostasis and/or thrombosis. *Commd7* and *Lrrfip1* transcripts on the other hand were identified based on their correlation with the platelet response toward ADP and/or CRP activation and the presence of SNPs within the gene that showed a possible association with either myocardial infarction or platelet response. Consequently, the contribution of these six genes to thrombosis and hemostasis was further screened in zebrafish using the laser-induced thrombosis model. 4 19

Morpholino oligonucleotides were used to create a successful knockdown in zebrafish and laser-induced thrombosis indicated that BAMBI promoted thrombus formation. In the absence of BAMBI, thrombus formation was reduced, as the time at which the first thrombocytes attached (TTA) to the injured vessel wall was significantly delayed and the thrombus size (TSA) was significantly decreased. 4 Similar results were found in the LRRC32 knockdown zebrafish 4 ; although the increase in TTA was only marginally significant compared with controls, the thrombocyte aggregation was clearly impaired in *Lrrc32* -downregulated zebrafish after laser-induced thrombosis. Some caution, however, was warranted as knockdown by one morpholino did affect the TTA, whereas another one affected the TSA, while none did both. The evidence for the involvement of ESAM was provided as knockdown of ESAM resulted in a significantly increased thrombus size (TSA). 4 Equally, DCBLD2 downregulation resulted in larger thrombi as compared with the controls using the laser-induced thrombosis model. Interestingly, this implies that both ESAM and DCBLD2 function as an intrinsic inhibitor of thrombocyte function. Morpholino-based silencing further indicated a modest role for COMMD7 and a significant contribution of LLRFIP1 in thrombus formation after laser injury. Antisense knockdown of COMMD7 resulted in a decrease in TSA, whereas knockdown of LLRFIP1 resulted in a significant delay in the TTA of the thrombocytes and a significant reduction in TSA. 19

Overall, functional genomics demonstrated BAMBI, LRRC32, COMMD7, and LRRFIP to be positive regulators and ESAM and DCBLD2 as negative regulators in thrombus formation in zebrafish.

## The Use of Mouse Models to Reveal the Function of Newly Identified Platelet Receptors

The hemostatic system in zebrafish, despite having encouraging similarities with mammals, remains genetically and systemically quite distant from the human situation and hence functional confirmation in higher species is warranted. 38 The (genetically engineered) mouse is most often used to study thrombosis in vivo due to its small size, high throughput, and similarity (∼99%) with the human genome. Even though the mouse is more similar to human than the zebrafish, data obtained in mice are not always perfectly translatable to human. With regard to the hemostatic system, there are differences in platelet receptor expression. The thrombin receptors are a known example for this as human platelets express PAR1 and PAR4, whereas murine platelets express PAR3 and PAR4. 39 Also, mice lack the equivalent of human FcγRIIa, while this receptor is important in binding the constant region of antibodies of the IgG class in human. 40 Murine platelets also fail to agglutinate in response to the antibiotic ristocetin, while this has a potent effect in human. Nevertheless, despite some differences, mouse models are indispensable to understand the fundamental mechanisms underlying diseases and to discover improved methods to prevent, diagnose, and treat them.

Thrombus formation in mice is mostly studied using either laser- or FeCl _3_ -induced injury of the mesenteric and carotid arteries or cremaster muscle arterioles. With laser-induced injury, the endothelial wall is damaged by heat, resulting in a controlled location and time of injury. The injury results in a fast platelet accumulation and expression of tissue factor at the injured vessel wall and is seen as inflammatory due to the absence of endothelial denudation. 41 This model, however, requires a lot of expertise and specialized equipment compared with the FeCl _3_ injury model, and therefore the latter can be applied in much more research facilities. Upon topical application of FeCl _3_ , the vessel denudates as a result of free radical formation causing lipid peroxidation and destruction of endothelial cells. 42 However, scanning electron microscopic analysis demonstrated that the endothelial cells are not denuded and that a thrombus is formed by erythrocyte-derived material mediating the binding of platelets to the endothelium. 43 The injury results in the expression of several adhesion molecules triggering leukocyte adhesion, platelet adhesion and aggregation, and increased tissue factor–dependent thrombus formation. The extent of thrombus formation, however, can be variable, and FeCl _3_ was shown to decrease platelet adhesion to several adhesion proteins. 42

With the discovery of the six platelet genes in 2009 and 2010, all have in the meantime been studied using in vivo murine models as described earlier and/or in ex vivo human platelet studies.

### BAMBI

The function of BAMBI as positive regulator of thrombus formation in zebrafish was confirmed by Salles-Crawley et al using *Bambi ^−/−^* mice that had an increased tail bleeding time, an impaired thrombus formation after FeCl _3_ injury of the mesenteric arterioles, and a decreased thrombus stability after laser-induced thrombosis. 44 However, unexpectedly, no defect in platelet behavior could be discerned ex vivo. Since BAMBI is completely deficient in both knockdown zebrafish and *Bambi ^−/−^* mice, the latter observation pointed into the direction of endothelial rather than platelet BAMBI to be responsible for the observed effects. Consequently, radiation chimeric mice were generated to investigate the contribution of endothelial versus platelet BAMBI. Radiation chimeric mice are prepared by irradiating recipient mice to kill the bone marrow and are next injected with hematopoietic progenitor cells from donor mice with a different genotype (here WT cells in KO mice or vice versa) that then repopulate the recipient bone marrow. Although thrombus size and time to reach maximal thrombus size did not differ, mice lacking BAMBI on the endothelial cells only demonstrated a decrease in thrombus stability compared with mice lacking BAMBI on their platelets. 44 This demonstrated that actually endothelial BAMBI rather than platelet BAMBI is involved in thrombus stability. Although different endpoints were determined in zebrafish (thrombus size) compared with chimeric mice (time to thrombus dissolution), both studies nevertheless concluded that BAMBI is a positive regulator of thrombus formation.

### LRRC32

In the knockdown zebrafish, LRRC32 was found to be a positive thrombosis regulator. 4 To further investigate this, we generated both platelet and endothelial-specific *lrrc32* knockout mice, as the full *lrrc32* knockout in mice was lethal. 45 This was done using the Cre-loxP recombination system in which the *Cre* gene was expressed after either the *Pf4* or the *Tie2* promoter. 45 Expression of LRRC32 on mouse platelets and endothelial cells was demonstrated by flow cytometry, by which we could confirm the absence of LRRC32 on either platelets or endothelial cells in the specific knockouts. 45 46 47 48 However, no differences were observed using either FeCl _3_ -induced thrombosis models in the carotid and mesenteric artery or in the tail bleeding time in platelet- or endothelial-specific *lrrc32* -deficient mice compared with littermate controls. 45 Additionally, using in vitro activation and aggregation experiments, no differences could be detected between platelets obtained from platelet-specific *lrrc32* knockout and control mice. Therefore, the role of LRRC32 as a positive regulator of thrombosis, as suggested in zebrafish, could not be confirmed in mice, at least for as far as LRRC32 on platelets and endothelial cells is concerned.

Although the function of platelet LRRC32 thus remained elusive, Rachidi and colleagues, however, recently reported that conditional *garp/lrrc32* deletion in platelets improves adoptive T-cell therapy as tumor treatment in mice. 46 Therefore, the role of platelet LRRC32 may perhaps be more related to inflammation and immune regulation, rather than to thrombosis and hemostasis, as was suggested in the zebrafish model.

### ESAM

In the same year as the zebrafish study, corroborating results in mice were published by Stalker and colleagues who demonstrated that platelet ESAM is involved in the interplatelet interaction due to its localization toward junctions between activated platelets. 49 50 Furthermore, genetic ablation of *Esam* resulted in more stable and larger thrombi upon laser injury of the cremaster muscle arterioles compared with controls. 49 To prove that the observed effect in the full knockout mice was indeed due to the expression of ESAM on platelets, radiation chimeric mice were generated using transplanted fetal liver cells. Mice lacking ESAM on their platelets, but with unaltered endothelial ESAM expression, again formed larger thrombi than mice expressing ESAM only on their platelets. 49 Accordingly, a similar enhancing effect of ESAM deficiency was demonstrated in in vitro aggregations of platelets from *Esam* ^−/−^ mice. 49

In humans, increased levels of soluble ESAM are associated with major cardiovascular risk factors and inflammatory markers. 51 Furthermore, aortic sinus lesions were significantly smaller and macrophage infiltration was reduced in the atheroma in *Esam ^−/−^ apoE* ^−/−^ mice compared with *Esam ^+/+^ apoE* ^−/−^ mice. 52 At last, ESAM expressed on endothelial cells, but not on platelets, is involved in neutrophil extravasation, as it has a destabilizing effect on the tight junctions. 53 Altogether, these results confirm that ESAM has an inhibitory effect on the formation of thrombi and suggest that ESAM is involved in atherosclerosis; however, the role of platelet ESAM in the latter remains unclear.

### DCBLD2

**Fig. 1:**
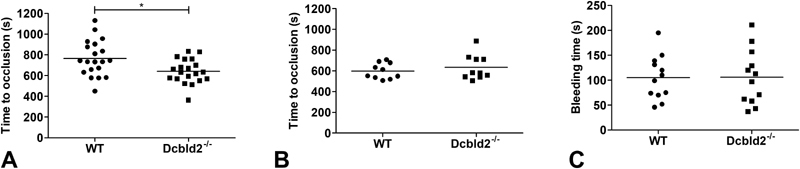
In vivo models: ( **A** ) Mesenteric arteries of C57Bl/6 or *Dcbld2* ^*−/−*^ mice were exposed and damaged by topical application of 10% FeCl _3_ for 2 minutes. Platelets were visualized using fluorescence microscopy after retro-orbital injection of rhodamine 6G. **(B** ) Carotid artery blood flow in C57Bl/6 or *Dcbld2 ^−/−^* mice following 3-minute exposure to 12% FeCl _3_ was monitored and the time needed for the vessel to occlude was determined. ( **C** ) Tail clipping assay was performed by cutting 2 mm of the mouse tail and immersing it in physiological water at 37°C. The time until the bleeding stopped was measured. Error bars represent mean ± SEM.

The evidence for the involvement of DCBLD2 in thrombosis was first provided by the zebrafish laser-induced thrombosis model where DCBLD2 functions as a negative regulator of thrombosis. The role of DCBLD2 in mice was further investigated in our group using *Dcbld2 ^−/−^* mice. The *Dcbld2 ^−/−^* mice showed no apparent spontaneous bleeding or thrombotic events over their lifespan, and no significant difference in number of circulating platelets was measured between the *Dcbld2 ^−/−^* mice and *Dcbld2 ^+/+^* control mice. Platelets of *Dcbld2 ^−/−^* mice did not demonstrate abnormalities, as the expression levels of physiologically important platelet receptors such as GPIb, integrin αIIb, and GPVI were not altered on platelets at rest or after activation by thrombin. Different in vivo bleeding and thrombosis models were used to perform experiments in *Dcbld2 ^−/−^* mice; bleeding time was determined by the tail clipping assay, and time to artery occlusion upon FeCl _3_ injury was determined in the large carotid artery and in the smaller mesenteric artery using a milder lower FeCl _3_ concentration. Where only a modest reduction in thrombus formation was seen in the smaller mesenteric arteries using a milder injury ( Fig. 1A ), no effect on thrombus formation in the large carotid artery ( Fig. 1B ) or on the bleeding time ( Fig. 1C ) was observed.

**Fig. 2:**
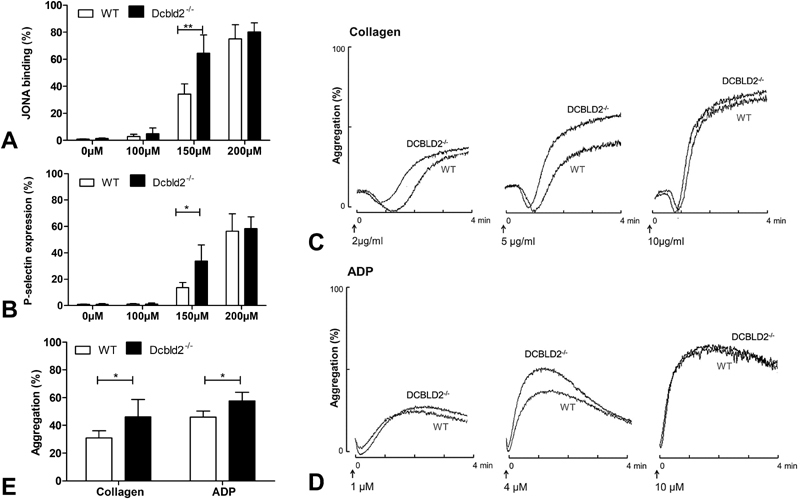
Lack of DCBLD2 increased platelet activation and aggregation using suboptimal agonist concentrations. Platelet-rich plasmas (PRP) from C57Bl/6 and *Dcbld2 ^−/−^* mice were used to activate and aggregate the platelets. ( **A, B** ) Platelets activated with different concentrations of PAR4-AP ranging from 0 to 200 μM. Activation of the αIIbβ3 receptor ( **A** ) and surface expression of P-selectin ( **B** ) was measured using flow cytometry. Platelet aggregation was induced using different concentrations of collagen ( **C** ) and ADP ( **D** ) at low, suboptimal, and high concentrations. Arrows indicate the time points of agonist addition. ( **C** and **D** ) Representative aggregation curves. ( **E** ) Quantitative analysis of aggregations induced with 5 μg/mL collagen or 4 μM ADP. Values are mean ± SEM from at least three independent experiments.

In agreement with the subtle shortened time to occlusion in the mesenteric thrombosis model was the observation that platelets obtained from *Dcbld2 ^−/−^* mice could be more readily activated. This was seen as an increased aggregation response in vitro, but only when the platelets were stimulated with suboptimal agonist levels ( Fig. 2A – E ).

In conclusion, these data identified DCBLD2 as a receptor that mildly downregulates platelet activation and aggregate formation at submaximal agonist concentrations. Although these effects are in line with the observations described earlier in the zebrafish model, 4 it is nevertheless nearly undetectable in mice and the relevance therefore remains doubtful.

### LRRFIP1

In the initial observation, LRRFIP1 expression in human platelets correlated positively with the level of fibrinogen binding in response to ADP. 19 This was supported by the significantly reduced ability of thrombocytes to support thrombus formation in the LRRFIP1-morphant zebrafish. 19 In line with this, silencing of LRRFIP1 by lentiviral transfection of shRNA into murine bone marrow cells used to generate radiation chimeric mice did indeed result in an antithrombotic effect in a deep vein thrombosis model. 54 Although the exact mode of action of LRRFIP1 remains unclear, as LRRFIP1 interacts with Flightless 1 (FLI1) and Debrin 1 (DBN1), both involved in the modulation of the cytoskeleton, it is possible that LRRFIP1 may contribute to cytoskeletal regulation of platelet activation. 19 Furthermore, LRRFIP1, through its effects on β-catenin, may also modulate canonical Wnt signaling, which was identified as a negative regulator of platelet function. 55

### COMMD7

Although a significant decrease in thrombus area was found in zebrafish with the downregulated *commd7* gene, no further in vivo research was done concerning thrombosis and cardiovascular effects. However, initially, the Bloodomics studies identified a correlation of transcript levels of COMMD-7 with variation in the human platelet response. More in particularly, snp rs6141803 of COMMD7 was found to correlate with cardiovascular disease. 19 This was later on confirmed independently. 56 57 The overall picture seems to indeed strengthen the assignment for COMMD7 as a contributor to thrombosis. Nevertheless, how COMMD7, so far only known as a repressor of transcription, might affect platelet function awaits further study.

## Conclusion

**Fig. 3:**
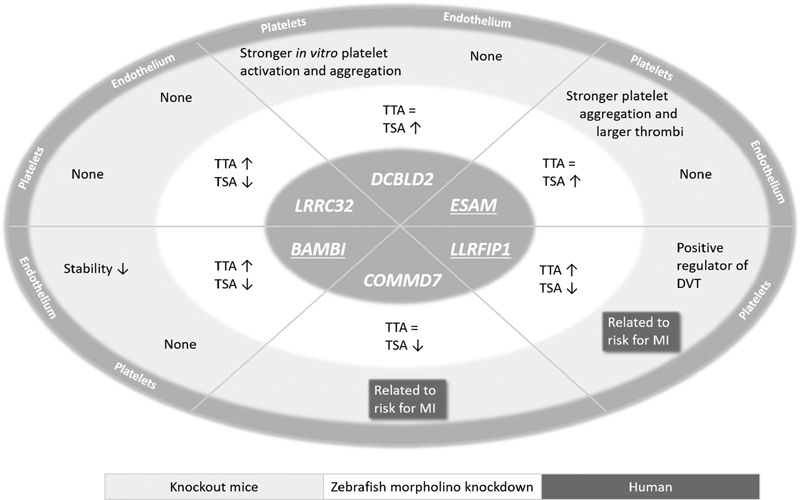
Thrombus formation after gene silencing in zebrafish and mice. Zebrafish: time to attachment (TTA) and thrombus surface area (TSA) were measured in gene-silenced zebrafish after laser-induced thrombosis. Mice: in vitro and in vivo aggregation studies were performed in transgenic mice. DVT, deep venous thrombosis. Human: association of SNPs in human genes for myocardial infarction (MI).

Six genes were analyzed in the Bloodomics studies as putative important mediators of platelet activation using a laser injury thrombosis model in gene-silenced zebrafish ( Fig. 3 ). 4 19

For two of them, COMMD7 and LRRFIP1, the initial selection was based on association with altered human platelet activation signals, which could be confirmed for LLRFIP1 in both silenced zebrafish and in radiation chimeric mice. Knockdown of COMMD7 had only a modest effect in zebrafish, whereas no data in mice are available yet. The four other genes, showing increased expression in MKs and HUVECs, were encoding for putative receptors. Based on the zebrafish data, both ESAM and DCBLD2 were proposed to be negative regulators of thrombus formation, which indeed was already published in the same year for ESAM in mice. 49 However, in mice, only modest effects of full DCBLD2 KO could be observed in vitro and in the in vivo FeCl _3_ mesenteric artery thrombosis model. Silencing of *bambi* and *lrrc32* in the zebrafish thrombosis model indicated a positive modulatory function of these genes. The observed effect of LRRC32 could, however, not be reproduced in mice. 45 This is in contrast to BAMBI, which was essentially due to the presence of BAMBI on endothelial cells rather than on platelets. 44

Overall, although high hopes were raised with the availability of the omics technology to find novel regulators of hemostasis, the expectations were only partially fulfilled. In the platelet field, a first approach was to identify platelet-specific transcripts as potential leads toward relevant players. This valid approach has, however, inherently a high risk of selecting non–thrombosis-linked targets as platelets are not only involved in thrombosis and hemostasis but also in (thrombo-)inflammation, tumorigenesis, and angiogenesis. Indeed, currently platelets LRRC32 and DCBLD2, both of which were demonstrated not to be involved in hemostasis in mice, have been implicated in the control of T-cell immunity and angiogenesis, respectively. 17 46 The second approach started from a phenotypic identification by studying transcript expression in the healthy population in correlation with in vitro platelet reactivity parameters likely involved in thrombosis, hence enhancing the chance for success. This was followed by a further association study with SNPs involved in myocardial infarction and platelet response. Currently, the main approach seems to start from the diagnosis of hereditary bleeding or platelets disorders of unknown origin followed by other strategies in genomics such as high-throughput sequencing, in the hope to identify the culprit. 58

Different platforms are introduced in the past few years to identify the underlying cause of inherited platelet disorders using next-generation sequencing. In the BRIDGE-Bleeding and Platelet Disorders (BPD) study, novel variants in different genes, such as *SRC* and *ACTN1* genes, were found to cause bleeding using high-throughput sequencing of a large patient cohort and the human phenotype ontology. 59 The Genotyping And Phenotyping of Platelets (GAPP) study combines whole-exome sequencing with platelet phenotyping to identify the causative genes in patients with unexplained bleeding disorders. Using this method, variants in *SLFN14* , *RUNX1* , and *FLI1* genes were found to be involved in bleeding. 60 61 Also the ThromboGenomics platform has used whole-genome sequencing to further expand the number of genes and their variants, such as *RASGRP2* , involved in inherited bleeding disorders. 62

This demonstrates that genomics underwent an evolution in which initially platelet-specific genes were identified and investigated, followed by the identification of genes whose transcripts correlated with a phenotype, to currently end up starting with a diagnosed platelet defect. This approach pretty much resembles the one that in the early days led to the discovery of GPIb and αIIbβ3. Nevertheless, starting from the diagnosis of hereditary bleeding or platelet disorders provides much more power to provide insight into platelet function and to unravel new platelet receptors with a putative role in thrombosis and hemostasis.
